# CD44 Promotes Migration and Invasion of Docetaxel-Resistant Prostate Cancer Cells Likely via Induction of Hippo-Yap Signaling

**DOI:** 10.3390/cells8040295

**Published:** 2019-03-30

**Authors:** Chih-Jen Lai, Ching-Yu Lin, Wen-Ying Liao, Tzyh-Chyuan Hour, Horng-Dar Wang, Chih-Pin Chuu

**Affiliations:** 1 Institute of Cellular and System Medicine, National Health Research Institutes, Miaoli 35053, Taiwan; krad0127@gmail.com (C.-J.L.); cylin071@nhri.org.tw (C.-Y.L.); 2 Institute of Biotechnology, National Tsing Hua University, Hsinchu City 30013, Taiwan; 3 Nation Institute of Cancer Research, National Health Research Institutes, Miaoli 35053, Taiwan; wyliao777@gmail.com; 4 Department of Biochemistry, Kaohsiung Medical University, Kaohsiung City 80708, Taiwan; 5 Department of Medical Research, Kaohsiung Medical University, Kaohsiung City 80708, Taiwan; 6 Department of Life Science, National Tsing Hua University, Hsinchu City 30013, Taiwan; 7 Graduate Institute of Basic Medical Science, China Medical University, Taichung 40402, Taiwan; 8 Ph.D. Program for Aging, China Medical University, Taichung 40402, Taiwan; 9 Biotechnology Center, National Chung Hsing University, Taichung 40227, Taiwan

**Keywords:** prostate cancer, CD44, YAP, docetaxel-resistant, migration, invasion, PC-3, DU-145

## Abstract

Patients receiving docetaxel developed a drug resistance within a few months. We generated docetaxel-resistant PC/DX25 and DU/DX50 CRPC cells from PC-3 and DU-145 PCa cells, respectively. We investigated the mechanism behind why PC/DX25 and DU/DX50 cells exhibited higher migration and invasion ability. Transwell assays were used to measure the migration and invasion of PCa cell. Fluorescence activated cell sorter (FACS) analysis was used to determine the population of cancer stem cell (CSC)-like cell. Micro-Western Array (MWA) was used to study the changes of the protein profile. FACS analysis revealed that PC/DX25 cells and DU/DX50 cells contain higher CD44+ population. MWA and Western blotting assay revealed that protein expression of CD44, YAP, CYR61, CTGF, phospho-ERK1/2 T202/Y204, ERK and vimentin was elevated in PC/DX25 cells. Knockdown of CD44 or YAP suppressed migration and invasion of PC/DX25 and DU/DX50 cells. Knockdown of CD44 decreased expression of YAP, CTGF and CYR61 but increased phosphorylation of S127 on YAP. CD44 knockdown also suppressed protein level of AKT, phospho-AKT T308, phospho-ERK1/2 T202/Y204 and vimentin. CD44 promotes migration and invasion of docetaxel-resistant PCa cells probably via induction of Hippo-Yap signaling pathway and CD44/YAP pathway may be a therapeutic target for docetaxel-resistant PCa.

## 1. Introduction

Prostate cancer (PCa) is the second most frequently diagnosed cancer of men and the fifth most common cancer overall in the world. Bones and lymph nodes are the most common metastatic sites for PCa. More than 80% of patients died from PCa developed bone metastases. Androgen ablation therapy is the primary treatment for metastatic PCa. However, a majority of PCa patients receiving the androgen ablation therapy will ultimately develop recurrent castration-resistant prostate cancer (CRPC) within one to three years after treatment with a median overall survival time of one to two years after relapse [1,2]. Chemotherapy is usually applied for treatment of metastatic CRPC [3].

Docetaxel belongs to the chemotherapy drug class taxane, and is being used to treat a variety of cancers, including breast cancer, head and neck cancer, stomach cancer, non-small-cell lung cancer, and PCa. Docetaxel is currently the most effective chemotherapy drug for CRPC metastatic PCa. Docetaxel treatment combined with prednisone or estramustine increased survival, reduced pain and serum PSA level, as well as increased the quality of life [4,5]. However, PCa patients receiving docetaxel developed a resistance against docetaxel within a few months. The mechanisms for the docetaxel-resistance in PCa cells include the expression of class III β-tubulin [6], elevation of AKT-dependent drug transporter ABCB1 (MDR-1) [7], reduction of PTEN and induction of c-Myc [8]. We have generated docetaxel-resistant PC-3 cells (named PC3/DX25) [7] and DU-145 cells (named DU/DX50). We observed that these docetaxel-resistant PCa cells migrate and invade much faster than their parental cell line, which may contribute to the aggressiveness of docetaxel-resistant PCa cells. We therefore applied Micro-Western Array, a high-throughput antibody-based proteomic platform [9], to investigate the mechanism for the higher metastatic potential of docetaxel-resistant PCa cells. Our study suggested that CD44 promotes migration and invasion of docetaxel-resistant PCa cells via induction of Hippo-Yap signaling.

## 2. Results

### 2.1. Docetaxel-Resistance Prostate Cancer (PCa) Cells Acquired Higher Migration and Invasion Ability than Parental PCa cells

**Figure 1:**
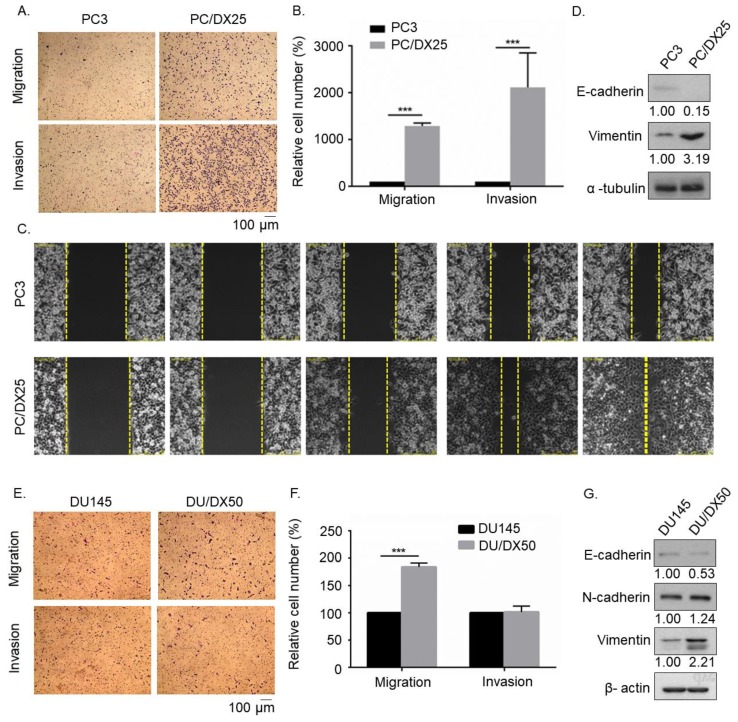
Transwell assay and wound healing assay revealed that docetaxel-resistant PCa cells exhibited higher migration and invasion ability as compared to parental PCa cells. (**A**) Images of PC-3 and PC/DX25 cells in migration and invasion transwell assays. (**B**) Quantification of cell migration and invasion of PC3 cells and PC/DX25 cells in (A). (**C**) Cell mobility of PC-3 and PC/DX25 cells was determined by wound healing assay at 0, 4, 8, 12, 16 h after the scratching. (**D**) Protein expression level of E-cadherin and vimentin was determined by Western blotting. The α-tubulin was used as loading control. (**E**) Images of DU-145 and DU/DX50 cells in migration and invasion transwell assay. (**F**) Quantification of cell migration and invasion of DU-145 cells and DU/DX50 cells in (E). (**G**) Protein expression level of E-cadherin and vimentin was determined by Western blotting. The β-actin was used as loading control. Asterisk *** represents statistically significant difference of *p* value < 0.001.

To compare the migration and invasion ability of docetaxel-resistant PCa cells and their parental PCa cells, we performed transwell migration and invasion assay (Figure 1A,B) as well as wound healing assay (Figure 1C). PC/DX25 cells migrated and invaded much faster than the parental PC-3 cells. Western blotting assay revealed that PC/DX25 cells expressed higher vimentin protein level, but lower of E-cadherin protein level (Figure 1D). Docetaxel-resistant DU/DX50 cells also exhibited higher migration ability than parental DU-145 cells (Figure 1E,F), although the difference in invasion ability was not significant. In DU/DX50 cells, protein level of vimentin was also higher but E-cadherin protein level was lower (Figure 1G). These data suggested that docetaxel-resistant PCa cells were more malignant than parental PCa cells.

### 2.2. Docetaxel-Resistant PCa Cells Contain Higher CD44+ Population

**Figure 2:**
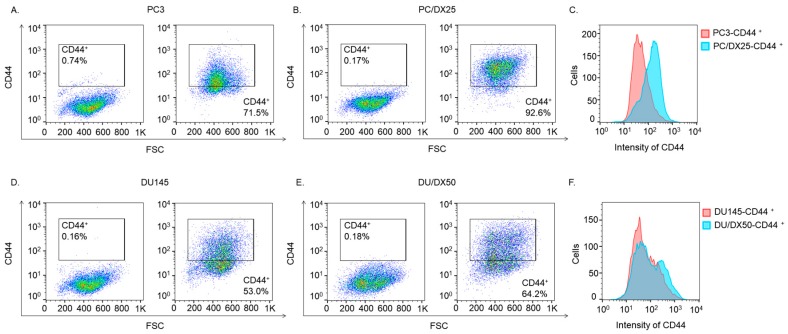
FACS analysis of CD44 protein expression in PC-3, PC/DX25, DU-145, DU/DX50 cells. Fluorescence activated cell sorter (FACS) analysis was used to analyze the CD44+ and CD44− populations in PC-3 (**A**), PC/DX25 (**B**) cells using PE filter (**C**), as well as to analyze the CD44+ and CD44− populations in DU-145 (**D**), DU/DX50 (**E**) cells using PE filter (**F**).

As cancer stem cell (CSC) has been reported to enhance aggressiveness and metastasis of cancer cells, we used fluorescence activated cell sorter (FACS) analysis to determine the populations of CD44+ cells in docetaxel-resistant PC/DX25 cells and DU/DX50 cells as well as their parental PC-3 and DU-145 cells. PCa cells with elevated CD44-positive [10] have been shown to acquire CSC-like characteristics. Compared to parental PC-3 cells and DU-145 cells, the PC/DX25 cells (Figure 2A–C) and DC/DX50 cells (Figure 2D–F) contain relatively higher CD44+ population. These observations indicated that PC/DX25 cells contain higher percentage of CSC-like population.

### 2.3. Docetaxel Resistant PCa Cells Express Higher Level of Proteins Involved in Hippo-YAP Pathway

**Figure 3:**
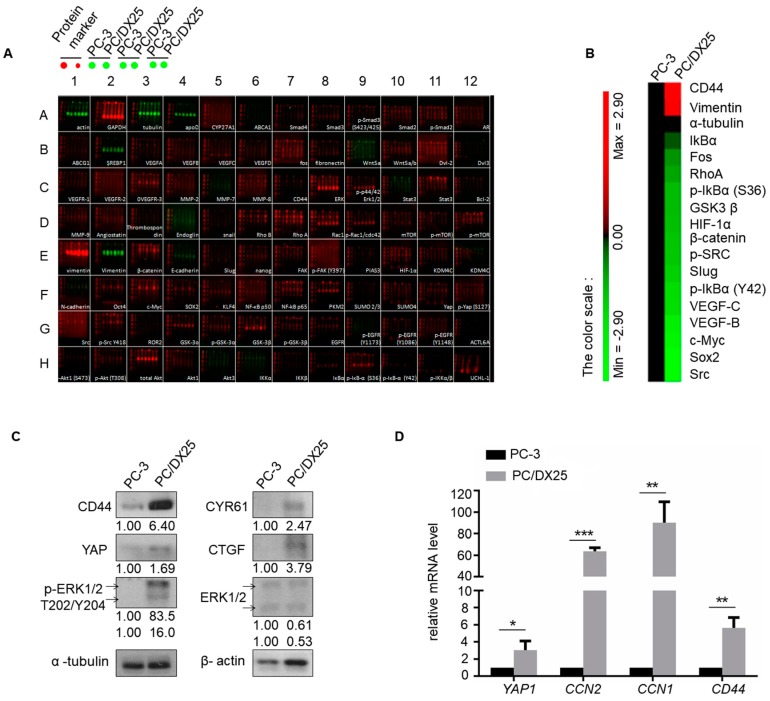
Profile of proteins regulating cell migration and invasion in PC3 vs. PC/DX25 cells as determined by Micro-Western Array and Western blotting. (**A**) Expression of proteins regulating cell migration and invasion in PC-3 cells and PC/DX25 cells was determined by Micro-Western Array (MWA) with 192 different antibodies. A representative image MWA was shown. (**B**) Results of MWA in (A) were demonstrated by heatmap. (**C**) Protein expression of CD44, YAP, CYR61, CTGF, phospho-ERK1/2 T202/Y204 and ERK in PC-3 and PC/DX25 cells was determined by Western blotting assay. The α-tubulin was used as loading control. (**D**) Gene expression of *YAP1*, *CCN2* (gene of CTGF), *CCN1* (gene of CYR61) and *CD44* was determined by real-time quantitative PCR. Asterisks *, **, *** represent statistically significant difference of *p* value < 0.05, *p* value < 0.01, and *p* value < 0.001, respectively.

Signaling proteins involved in Hippo-YAP pathway have been reported to regulate stemness and cancer metastasis, we therefore performed Micro-Western Array (Figure 3A,B) and Western Blotting (Figure 3C) to compare the profile of signaling proteins in docetaxel-resistant PC/DX25 and PC-3 cells. MWA and Western blotting revealed that protein expression level of CD44, Yes associated protein 1 (YAP), Cysteine Rich Angiogenic Inducer 61 (CYR61), Connective Tissue Growth Factor (CTGF), phospho-ERK1/2 Thr202/Tyr204, and ERK was higher in PC/DX25 cells as compared to their parental PC-3 cells. In addition, real-time quantitative PCR showed that PC/DX25 cells express higher mRNA level of YAP1, CTGF, CYR61, and CD44 (Figure 3D).

### 2.4. Knockdown of CD44 or YAP Suppresses Migration and Invasion of Docetaxel-Resistant PCa Cells

**Figure 4:**
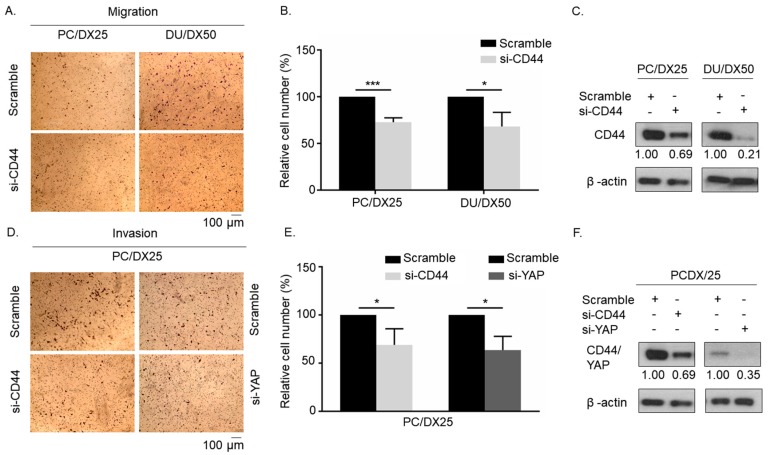
Knockdown of CD44 or YAP protein suppresses cell mobility of docetaxel-resistant PCa cells. (**A**) Migration of PC/DX25 and DU/DX50 cells with scramble control or CD44 siRNA knockdown was determined by transwell migration assay. (**B**) Quantification of cell migration of PC/DX25 and DU/DX50 cells with scramble control or CD44 siRNA knockdown in (**A**). (**C**) Confirmation of CD44 knockdown in PC/DX25 and DU/DX50 cells with Western blotting. (**D**) Invasion ability of PC/DX25 cells with CD44 siRNA knockdown or YAP siRNA knockdown was compared to PC/DX25 cells with scramble control using transwell invasion assay. (**E**) Quantification of cell invasion of PC/DX25 cells with or without CD44 siRNA knockdown or YAP siRNA knockdown in (**D**). (**F**) Confirmation of CD44 and YAP knockdown in PC/DX25 cells with Western blotting. Asterisks *, *** represent statistically significant difference of *p* value < 0.05, and *p* value < 0.001, respectively.

To investigate if CD44 regulates cell mobility of docetaxel-resistant PCa cells, we knocked down CD44 with siRNA in PC/DX25 and DU/DX50 cells. Transwell migration assay revealed that knockdown of CD44 significantly suppressed the cell migration of PC/DX25 and DU/DX50 cells as compared to the scramble control (Figure 4A–C). Additionally, siRNA knockdown of either CD44 or YAP protein significantly suppressed the invasion of PC/DX25 cells (Figure 4D–E). These results suggested that both CD44 and YAP proteins play an important role in the regulation of cell migration and invasion of docetaxel-resistant PCa cells.

### 2.5. CD44 Regulated the Expression of YAP in Docetaxel-Resistant PCa Cells

**Figure 5:**
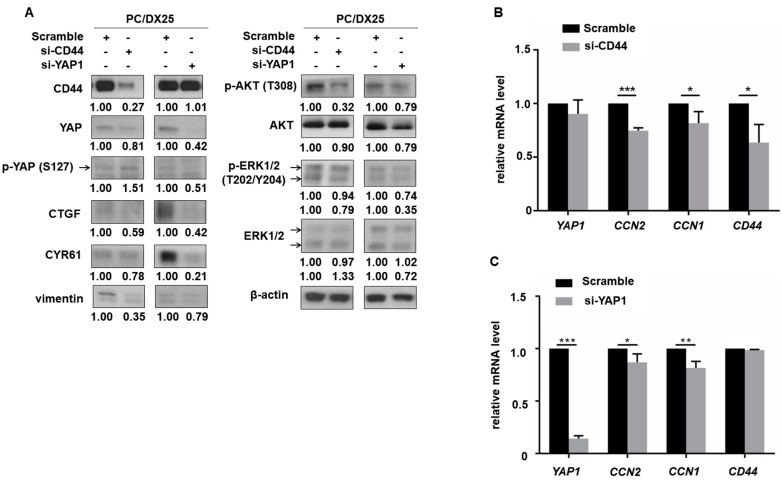
Knockdown of CD44 protein in docetaxel-resistant PCa cells inhibits Hippo-YAP signaling pathway. (**A**) Protein level of CD44, YAP, phospho-YAP (S127), CYR61, CTGF, phospho-AKT (T308), AKT, phospho-ERK1/2 (T202/Y204), ERK1/2 and vimentin in PC/DX25 cells with or without CD44 siRNA knockdown or YAP siRNA knockdown was determined by Western blotting. Expression of β-actin was used as loading control for all Western blots. (**B**) Gene expression level of *YAP1*, *CCN2*, *CCN1*, and *CD44* in PC/DX25 cells with CD44 siRNA knockdown or (**C**) PC/DX25 cells with YAP siRNA knockdown was examined by qRT-PCR. Asterisks *, **, *** represent statistically significant difference of *p* value < 0.05, *p* value < 0.01, and *p* value < 0.001, respectively.

To determine the relationship between CD44 and YAP in regulation of migration and invasion of the docetaxel-resistant PCa cells, we knocked down CD44 and YAP with siRNA individually in PC/DX25 cells. Knockdown of CD44 suppressed expression of YAP and its downstream target proteins CTGF and CYR61 (Figure 5A) but increased the protein level of phospho-YAP S127. Phosphorylation of S127 on YAP retains YAP protein in cytoplasm and thus decreases the oncogenic activity of YAP. On the other hand, knockdown of YAP with siRNA did not affect the protein level of CD44, suggesting that YAP is downstream of CD44 in PCa cells. Knockdown of YAP decreased the protein level of CTGF and CYR61 (Figure 5A). Additionally, knockdown of either CD44 or YAP reduced the protein expression of AKT, phospho-AKT T308, phospho-ERK1/2 T202/Y204 and vimentin (Figure 5A). Interestingly, knockdown of CD44 did not affect level of *YAP1* gene as determined by qRT-PCR but suppressed gene level of *CCN2* (gene of CTGF) and *CCN1* (gene of CYR61) (Figure 5). Knockdown of YAP repressed gene level of *CCN2* and *CCN1*, but not gene level of *CD44* (Figure 5C). These observations suggested that the regulation of YAP by CD44 might be post-translational.

## 3. Discussion

In this study, we observed that docetaxel-resistant PC/DX25 and DU/DX50 CRPC cells exhibited higher migration and invasion ability. FACS analysis revealed that docetaxel-resistant PCa cells contain higher CD44+ population, implying that these cells have higher cancer stem cell population. Micro-Western Array and Western blotting assay showed that the protein expression of CD44, YAP, CYR61, CTGF, phospho-ERK1/2 T202/Y204, ERK, and vimentin in PC/DX25 cells was elevated. Knockdown of CD44 or YAP suppressed migration and invasion of PC/DX25 and DU/DX50 cells. CD44 knockdown decreased the expression of YAP and its downstream target proteins CTGF and CYR61, but increased phosphorylation of S127 on YAP. Knockdown of YAP decreased CTGF and CYR61 protein abundance, but not protein expression of CD44, suggesting that CD44 function as an upstream regulator of YAP. CD44 knockdown also suppressed protein level of AKT, phospho-AKT T308, phospho-ERK1/2 T202/Y204 and vimentin. Our study suggested that CD44 promotes migration and invasion of docetaxel-resistant PCa cells possibly via the induction of Hippo-Yap signaling.

Knockdown of CD44 or CD147 has previously been shown to decrease proliferation and invasion of docetaxel-resistant PC-3M-luc PCa cells, as well as suppressed tumorigenesis and cancer metastasis of PC-3M-luc cells in xenograft model [11]. Knockdown of CD44 or CD147 enhanced docetaxel sensitivity and decreased abundance of phospho-AKT and phospho-ERK [11]. CD44 is a cell surface glycoprotein involved in cell adhesion, migration, drug resistance, and signal transmission [12,13]. CD44 is the receptor for hyaluronic acid (HA), a major component of the extracellular matrix (ECM). HA-CD44 interaction activates gene expression of STAT-3-mediated multidrug resistance protein 1 (MDR1) and stemness gene Nanog [14]. Elevation of CD44 has been observed in some other drug-resistant cancer cell lines. For example, fluorouracil-resistant colon cancer cells express higher level of CD133 and CD44 proteins [15]. Breast cancer cells resistant to treatment of SAHA, a HDAC inhibitor, also express higher CD44 protein expression [16].

**Figure 6:**
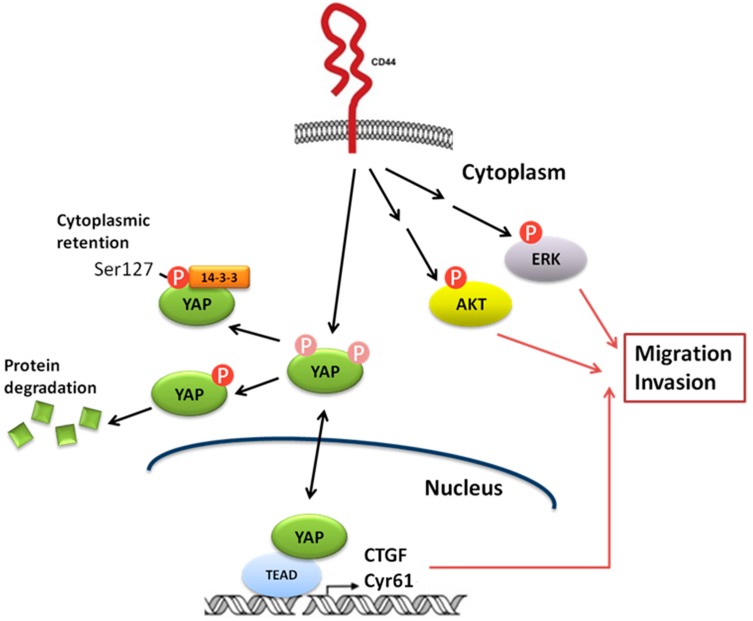
Proposed mechanism how CD44 proteins promotes migration and invasion of docetaxel-resistance PCa cells. Based on the observation in this study, we proposed that CD44 promotes migration and invasion of docetaxel-resistance PCa cells possibly through the activation of Hippo-YAP, ERK, and AKT signaling cascade.

Switching of CD44 splice isoform in breast cancer cells has been reported to activate AKT signaling, and is essential for epithelial-mesenchymal transition (EMT) and cancer progression [17]. CD44+ population of PCa cells exhibit cancer stem cell (CSC) characteristics [18] and correlate to poor survival of PCa patients [19]. AKT/PI3K signaling being activated by CD44 and CD133 is essential for maintenance of cancer stemness in PCa cells [20]. Hippo-YAP pathway has been discovered to regulate cell proliferation and apoptosis [21,22], and is essential in the regulation of cancer development [23]. Components of the Hippo pathway, including Yap, Lats1/2, and Mst1/2 are highly conserved during evolution [21,22]. Expression of YAP protein is frequently elevated in several types of cancer, including prostate cancer, and has been confirmed to be an oncogene [23,24]. LATS1 phosphorylates YAP on S127 which promotes the binding between YAP and 14-3-3, therefore sequestrates YAP in the cytoplasm and suppresses transcriptional activity of YAP [25]. Transcription factors from TEAD family are essential in mediating YAP-dependent gene expression, YAP-induced cell growth, oncogenic transformation, and epithelial-mesenchymal transition in cancer cells [26]. Connective tissue growth factor (CTGF) and cysteine-rich angiogenic inducer 61 (CYR61) have both been identified as a direct target gene of YAP and TEAD, and is important for cell growth [26,27]. Immunohistochemistry revealed that YAP expression is upregulated and hyperactivated in castration-resistant prostate tumors, while the overexpression of YAP promotes migration, invasion, and androgen-independent proliferation of PCa cells [28]. The miR-302/367/LATS2/YAP pathway is essential for maintenance of cancer stemness in PCa cells and promotes the development of castration resistance [29]. CD44 has recently been reported to function as an upstream regulator of ERK, AKT, and Hippo-YAP pathway [30,31]. Knockdown of CD44 reduced expression and nuclear localization of YAP as well as suppressed the expression of YAP downstream effector genes *CCN1* (gene of CTGF), *CCN1* (gene of CYR61) and EDN1 [32]. CD44 regulates YAP via RhoA [32]. Knockdown of either CD44 or YAP induced cell apoptosis as well as inhibited the cell proliferation, cell cycle progression and migration of lung cancer cells [32]. CD44 expression positively correlates to the expression of YAP in hepatocellular carcinoma (HCC) and high expression of CD44 or YAP correlates to worse pathology grade, increased vascular invasion and more severe liver cirrhosis [33]. There is a positive feedback loop involving CD44 and YAP as CD44 positively regulates YAP via PI3K/AKT pathway, while CD44 is regulated by YAP/TEAD [33] in HCC. Similarly, in malignant mesothelioma (MM) cells, YAP/TEAD activates CD44 transcription by binding to the CD44 promoter at TEAD-binding sites, while CD44 regulates Merlin phosphorylation and sequentially promotes YAP transcriptional co-activator [34]. YAP/CD44 axis confers cancer stemness in MM cells and therefore enhance resistance against chemotherapy [34]. Our current study indicated that CD44 enhances invasion of docetaxel-resistant PCa cells probably via regulation of Hippo-Yap pathway. Figure 6 demonstrates the proposed mechanism how CD44 proteins promotes migration and invasion of docetaxel-resistance PCa cells. 

## 4. Materials and Methods 

### 4.1. Chemicals

Docetaxel used in this research was purchased from Sigma-Aldrich (St. Louis, MO, USA).

### 4.2. Cell Culture

PC-3 and DU-145 cells were purchased from ATCC (Manassas, VA, USA). Docetaxel-resistant sublines PC/DX25 and DU/DX50 sublines was developed by chronically exposing PC-3 and DU-145 cells to progressively increased concentrations of docetaxel in by Prof. Tzyh-Chyuan Hour’s lab (Department of Biochemistry, Kaohsiung Medical University, Kaohsiung, Taiwan). PC-3 and DU-145 cells were maintained in RPMI-1640 medium contained 10% FBS, penicillin (100 U/mL), and streptomycin (100 μg/mL) at 37 °C with 5% CO_2_. PC/DX and DU/DX cells were maintained with 25 nM and 50 nM docetaxel, respectively.

### 4.3. Transwell Migration Assay

PC-3, PC/DX25, DU145, and DU/DX50 cells were examined for their migration ability using 24-well transwell dishes with a pore size of 8 mm (BD Biosciences, San Jose, CA, USA). Cells were seeded at a density of 1 × 10^4^ cells in 500 μL serum free RPMI medium. Cells were placed in the upper chamber, while of complete medium (1000 μL) was placed in the lower chamber. The cells were incubated at 37°C and 5% CO_2_ for 12 to 16 h. Cells were then fixed in iced methanol for 5 to 10 min and stained with hematoxylin for 15 to 30 min. Cotton-tipped swabs were used to remove cells on the upper side of the filters, and the filters were then washed with dH_2_O. A microscope was used to examine and count cells on the underside of the filters. Each condition was plated in triplicate for each experiment. All experiments were repeated for at least three times.

### 4.4. Transwell Invasion Assay

PC-3, PC/DX25, DU-145, and DU/DX50 cells were examined for their invasion ability using an invasion assay with Growth Factor Reduced BD BioCoat Matrigel invasion chambers (BD Biosciences, San Jose, CA, USA) according to the manufacturer’s instructions. PC-3, PC/DX25, DU-145, and DU/DX50 cells were placed in the upper chamber and seeded at a density of 1 × 10^4^ in serum free RPMI medium (500 μL). Complete medium (1000 μL) was placed in the lower chamber. The cells were incubated at 37 °C and 5% CO_2_ for 12 to 16 h. Cells were then fixed in iced methanol for five to 10 min and stained with hematoxylin for 15 to 30 min. Cotton-tipped swabs were used to remove the cells on the upper side of the filters, and the filters were washed with dH_2_O. A microscope was used to examine and count cells on the underside of the filters. Each condition was plated in triplicate for each experiment. All experiments were repeated for at least three times.

### 4.5. Wound Healing Assay

PC-3 and PC/DX25 cells were examined for their mobility using wound healing assay with ibidi culture insert (Applied Biophysics, Troy, NY, USA.) according to the manufacturer’s instructions. PC-3 and PC/DX25 cells were seeded at a concentration of 3.5 × 10^4^/100 μL into individual compartment of ibidi culture insert overnight. The culture plate was filled with RPMI complete medium and the ibidi culture inserts was then removed. A live cell imaging microscope (Leica AF 6000 LX, Leica, Wetzlar, Germany) was used to monitor and to take photograph for the migration of the cells once per two hours.

### 4.6. Flow Cytometry

For surface maker analysis, PC-3 cells and PC/DX25 cells (1 × 10^6^) were resuspended in 100 μL of staining buffer. Fluorescent-conjugated antibodies were added and cells were dissociated, antibody-labeled (1:100 dilution per 10^6^ cells), incubated for 30 min on ice and resuspended in Hanks’ Balanced Salt Solution (HBSS; Invitrogen, Waltham, Massachusetts, USA) containing 2% FBS and 10mM HEPES (Invitrogen). The PE-conjugated anti-CD44 antibody (BD Biosciences, San Jose, CA, USA) was used. Flow cytometry was done using a FACSCaliburTM flow cytometer (BD Biosciences). FlowJo software (Ver10.0, (BD) Becton, Dickinson and Company, Franklin Lakes, New Jersey, USA) was used to analyze the data.

### 4.7. Micro-Western Arrays (MWA)

Whole cell lysates of PC-3 and PC/DX25 cells were harvested to perform Micro-Western Arrays. The MWA were conducted to measure protein expression with 96 antibodies detecting proteins regulating cancer metastasis as described previously [9,35]. The α-tubulin was used as a loading control. Scanned images were obtained using the Odyssey Infrared Imaging System. The intensity of bands for different proteins was quantified with Image Studio Ver 5.2 software (Li-Cor Bioscienses, Lincoln, Nebraska, USA).

### 4.8. Real-Time Quantitative PCR

PC-3 and PC/DX25 cells transfected with small interfering RNA cells or control scramble primer were extracted for RNA with the RNeasy Mini Kit from Qiagen (Germantown, MD, USA) following the manufacturer’s instructions. The primer sequences were designed by Primer3, and the sequences were as follows: YAP forward: 5′-GGTGCCACTGTTAAGGAAAGG-3′ and reverse: 5′-GTGAGGCCACAGGAGTTAGC-3′; CTGF forward: 5′-TGGTGCAGCCAGAAAGCTC-3′ and reverse: 5′-CCAATGACAACGCCTCCTG-3′; Cyr61 forward: 5′-TTCTTTCACAAGGCGGCACTC-3′ and reverse: 5′-AGCCTCGCATCCTATACAACC-3′; CD44 forward: 5′- GGTTACATCTTTTACACCTTTTCTAC-3′ and reverse: 5′-GAATGTGTCTTGGTCTCTGGTAG-3′. Real-time quantitative PCR was performed by ABI-7500 (Thermo Fisher Scientific, Waltham, MA, USA).

### 4.9. Knockdown of CD44 or YAP with Small Interfering RNA

Human CD44 siRNA (On-TargetPlus Human CD44 (960) small interfering RNA (siRNA) SmartPool, L-009999-00-0005), Human YAP1 siRNA (On-TargetPlus Human YAP1 (10413) small interfering RNA (siRNA) SmartPool, L-012200-00-0005) and nonspecific targeting (On-TargetPlus Nontargeting Pool, D-001810-10-05) were purchased from GE Dharmacon (Lafayette, CO, USA). The transfection was performed using lipofectamine RNAiMax (Thermo Fisher Scientific) according to the manufacturer’s protocol. PC/DX25 cells were transfected with either CD44 siRNA, YAP siRNA or scramble control for 72 h and then collected for Western blot analysis validation, as well as collected for transwell migration and invasion assay.

### 4.10. Western Blot Analysis

Cells were lysed in SDS lysis buffer (240 mM Tris-acetate, 1% SDS, 1% glycerol, 5 mM EDTA pH 8.0) with DTT, protease inhibitors, and a cocktail of phosphatase inhibitors. Antibody against vimentin, and MCP-1 were purchased from Abcam (Cambridge, MA, USA). Antibodies detecting CD44 and phospho-YAP S127 were purchased from GeneTex, Inc. (Irvine, CA, USA). Antibodies against YAP, phospho-MST1/MST2 T183/T180, MST1, phospho-LATS1 S909, LATS1, CYR61, CTGF, phospho–AKT T308, AKT, and phospho–GSK3β S9, phospho-ERK1/2, ERK1/2 were purchased from Cell Signaling Technology (Danvers, MA, USA). The β-actin, GAPDH, and α -tubulin antibodies were purchased from Novus Biologicals (Littleton, CO, USA). Antibody against E-cadherin was purchased from BD (Franklin Lakes, NJ, USA). 

## 5. Conclusions

CD44 promotes the migration and invasion of docetaxel-resistant PCa cells as well as emhances the Hippo-Yap signaling pathway in these PCa cells. Targeting CD44 and Hippo-YAP pathway may therefore be a potential treatment for docetaxel-resistant PCa.
